# Identification of two independent X-autosome translocations in closely related mammalian (*Proechimys*) species

**DOI:** 10.1038/s41598-019-40593-8

**Published:** 2019-03-11

**Authors:** Willam Oliveira da Silva, Marlyson Jeremias Rodrigues da Costa, Julio Cesar Pieczarka, Jorge Rissino, Jorge C. Pereira, Malcolm Andrew Ferguson-Smith, Cleusa Yoshiko Nagamachi

**Affiliations:** 1 Centro de Estudos Avançados da Biodiversidade, Laboratório de Citogenética, ICB, Universidade Federal do Pará, Belém, Pará Brazil; 2 Cambridge Resource Centre for Comparative Genomics, Department of Veterinary Medicine, University of Cambridge, Cambridge, UK

## Abstract

Multiple sex chromosome systems have been described for several mammalian orders, with different species from the same genus sharing the same system (e.g., X_1_X_2_Y or XY_1_Y_2_). This is important because the translocated autosome may be influenced by the evolution of the recipient sex chromosome, and this may be related to speciation. It is often thought that the translocation of an autosome to a sex chromosome may share a common origin among phylogenetically related species. However, the neo-X chromosomes of *Proechimys goeldii* (2n = 24♀, 25♂/NFa = 42) and *Proechimys* gr. *goeldii* (2n = 16♀, 17♂/NFa = 14) have distinct sizes and morphologies that have made it difficult to determine whether they have the same or different origins. This study investigates the origins of the XY_1_Y_2_ sex chromosome determination system in *P. goeldii* (PGO) and *P*. gr. *goeldii* (PGG) and elucidates the chromosomal rearrangements in this low-diploid-number group of *Proechimys* species. Toward this end, we produced whole-chromosome probes for *P. roberti* (PRO; 2n = 30♂/NFa = 54) and *P*. *goeldii* (2n = 25♂/NFa = 42) and used them in comparative chromosomal mapping. Our analysis reveals that multiple translocations and inversions are responsible for the karyotype diversity of these species, with only three whole-chromosomes conserved between PRO and PGO and eight between PGO and PGG. Our data indicate that multiple sex chromosome systems have originated twice in *Proechimys*. As small populations are prone to the fixation of chromosomal rearrangements, we speculate that biological features of Rodentia contribute to this fixation. We also highlight the potential of these rodents as a model for studying sex chromosome evolution.

## Introduction

Reproductive isolation is an important step in the speciation process. Speciation mediated by geographic isolation has been well documented and is generally accepted^1–4^, but the differentiation process between spatially contiguous populations is complex and not well documented. One particular type of sympatric speciation - that mediated by chromosomal changes - has been observed in both plants^5^ and animals^6^.

Chromosomal rearrangements have long been discussed for their ability to reduce the fertility of heterozygous individuals^7^. They can also reduce gene flow by suppressing recombination between the rearranged and parental segments, extending the effects of gene isolation^6^.

An example of the role of chromosomal rearrangements in the process of lineage diversification was found among two populations of *Gasterosteus aculeatus* that show distinct chromosomal sex determination systems (XX/XY and X_1_X_2_Y/X_1_X_1_X_2_X_2_)^8^. The authors showed that males with the X_1_X_2_Y system presented different spine sizes and courtship behavior compared to males with the XY system, and that these differentiated characteristics were associated with the neo-X chromosome. The phenotypes present in X_1_X_2_Y individuals may have arisen after the origination of the neo-Y and accumulated on the neo-X chromosome during 1.5–2 Ma (million years ago), which points to reproductive isolation mediated by an autosomal-sexual chromosomal translocation between closely related species^8^.

The sex chromosomes of therian mammals have been relatively stable since their origin 160 Ma^9^. However, fusions between autosomes and sex chromosomes have been described for several mammalian orders^10–13^. A meta-analysis of multiple sex chromosome systems in mammals^10^ demonstrates that different species of the same genus (e.g., *Artibeus*, *Carollia*, *Gazella*, *Sorex*, *Taterillus*, *Aotus* and *Alouatta*) may share the same multiple chromosome sex system (X_1_X_2_Y or XY_1_Y_2_). Comparison of chromosome painting results and/or C- and G-banding patterns confirms that the same autosome is involved in the translocations of bats from genera *Carollia*^14^, *Artibeus*, *Uroderma*^11^, *Chiroderma*, *Vampyriscus* and *Mesophylla*^15^, and in Primates from genera *Aotus*^16^ and *Alouatta*^17^. However, independent origins for the X-autosomal translocations in species of the same genus are found in African pygmy mice^18^, which reportedly exhibit translocations (X.1)(Y.1), (X.7), (X.12), (X.15)(Y.15) and (X.16)^12^.

**Figure 1:**
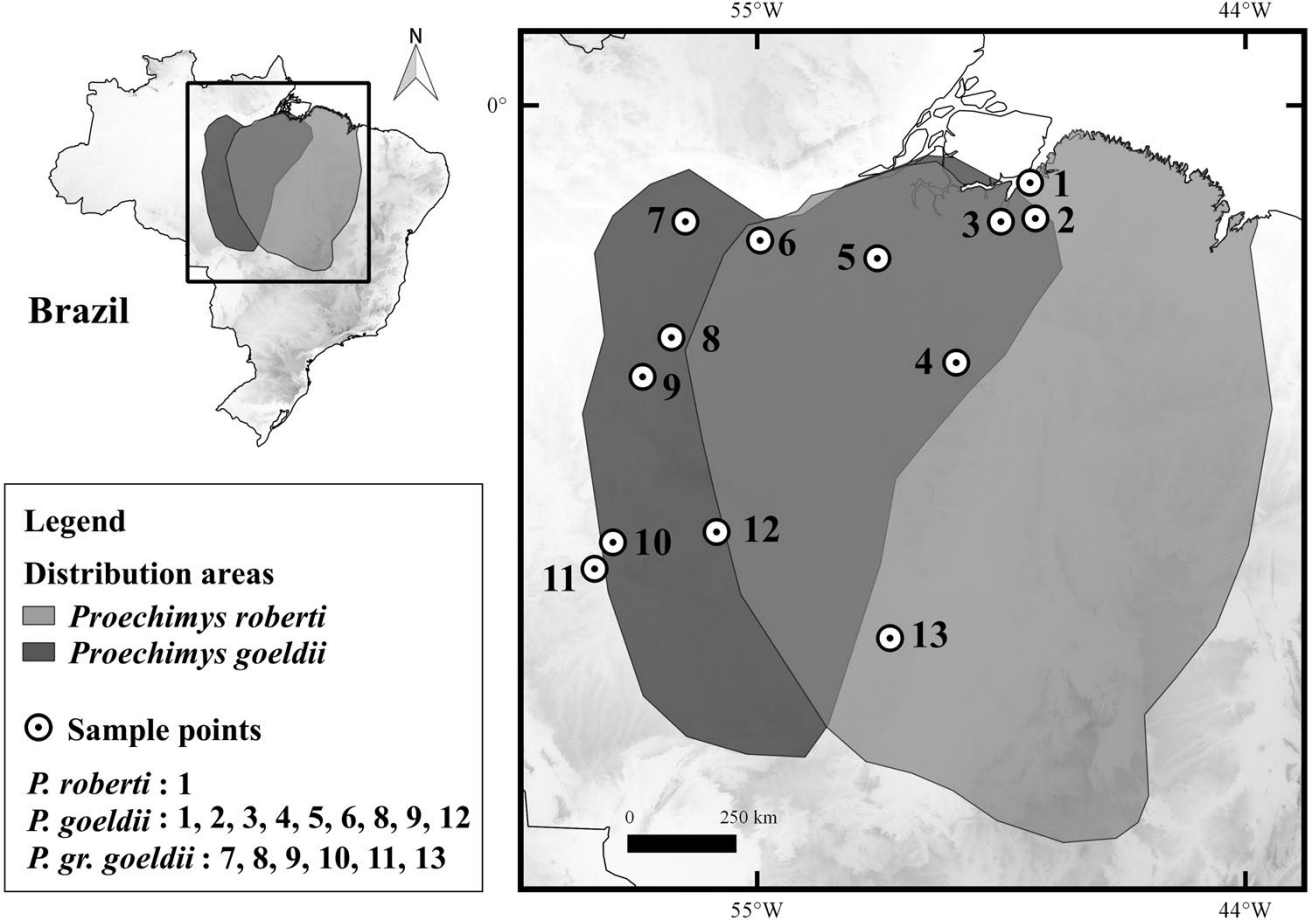
Map showing the distribution areas of *Proechimys roberti* and *P. goeldii*. The numbers refer to the collection points for the samples of *P. roberti*, *P. goeldii* and *P*. gr. *goeldii* karyotyped in the present study (localities 1 and 7) and in the literature (localities 2–6, 8–13), as detailed in Supplementary Table 1.

In the Brazilian Amazon, only two rodent species have been reported to exhibit multiple sex chromosomes, and both belonging to genus *Proechimys*: *Proechimys* gr. *goeldii* (two cytotypes with 2n = 14♀15♂ and 2n = 16♀17♂)^19–21^ and *Proechimys goeldii* (two cytotypes with 2n = 24♀25♂ and 2n = 26♀27♂)^22^ (Fig. 1, Supplementary Table 1). Although these two taxa share the same sex determination system (XY_1_Y_2_), their neo-X chromosomes have distinct sizes and morphologies and it is not known whether they have the same origin^22^.

This type of rearrangement (sex chromosome-autosome) is rare, and in humans and mice it has generally been associated with deleterious effects that compromise carrier fertility by inactivating the autosomal segment that is translocated to the sex chromosome^23–25^. Bats of genus *Carollia*^26^, however, have an XY_1_Y_2_ system in which the autosomal portion is not inactivated, apparently because a Nucleolar Organizer Region (NOR) located between the X and autosomal segments stops the spread of chromatin inactivation. A similar proposition has been made for bats of genera *Artibeus* (XY_1_Y_2_) and *Uroderma* (Neo-XY), that have a heterochromatic block instead of an NOR between the X and autosomal segments^27^. In Bovidae^13^, Fluorescence *in situ* Hybridization (FISH) and meiotic analyses reveal great diversity in chromosome size and morphology due to the presence of inversions, heterochromatin blocks and centromere shifts. Heterochromatin blocks between the fused sex chromosome and autosome have been proposed to suppress the spread of inactivation into the autosomal portion^13^.

Here, we used chromosome painting to investigate the XY_1_Y_2_ sex chromosome systems in *Proechimys goeldii* (2n = 24♀25♂/NFa = 42) and *Proechimys* gr. *goeldii* (2n = 16♀17♂/NFa = 14), and to determine if they have the same origin. We discuss possible causes for the establishment of this system and address two hypothetical scenarios in which sex chromosome-autosome rearrangements could play a crucial role in the speciation process, whether in allopatry or sympatry, for these two closely related species.

## Results

### Flow karyotyping and FISH assignment

**Figure 2:**
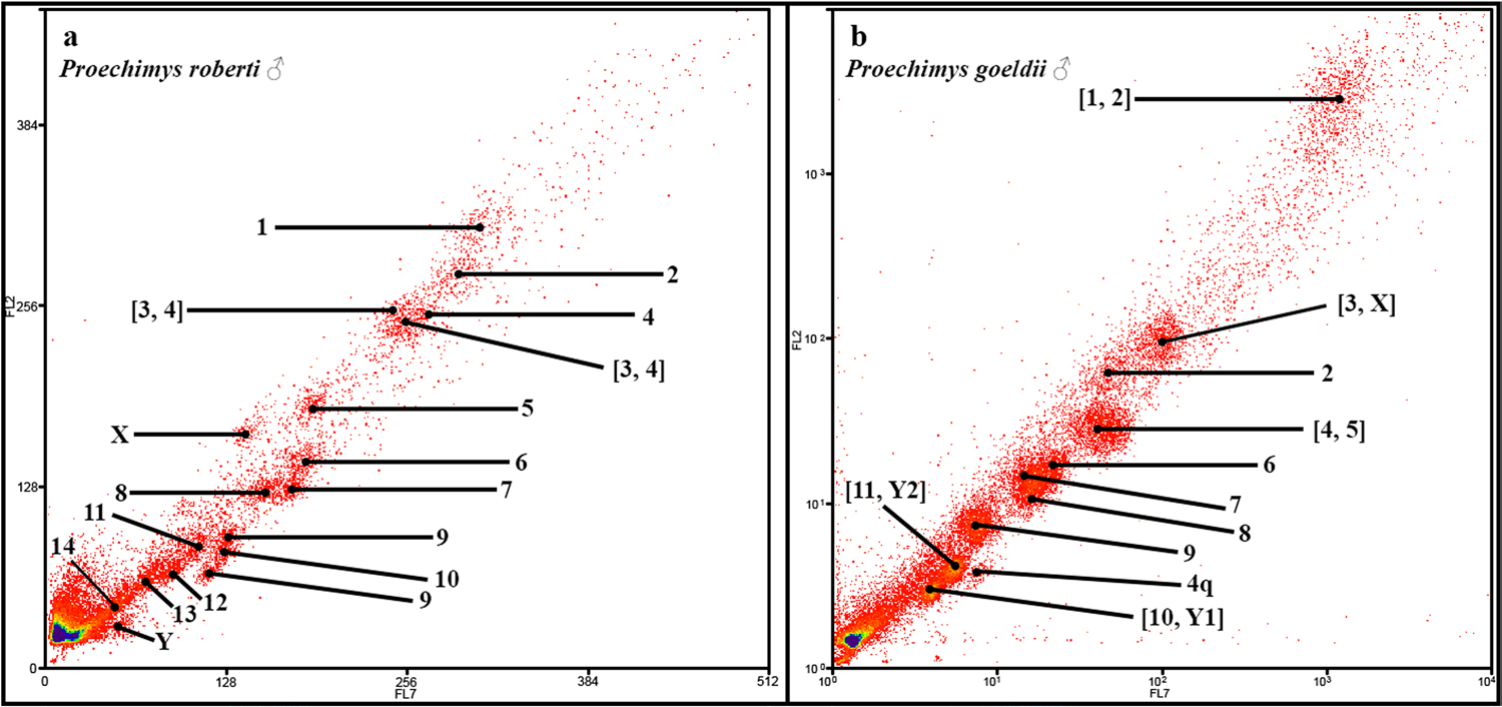
Flow karyotypes of (**a**) *Proechimys roberti* (PRO, 2n = 30♂) and (**b**) *P. goeldii* (PGO, 2n = 25♂).

*Proechimys roberti* (PRO) has a 2n = 30/NFa = 54 karyotype, with 13 meta/submetacentric pairs (1–13) of autosomes and one acrocentric pair (14). The X chromosome is a middle-sized submetacentric and the Y chromosome is a small acrocentric. Whole-chromosome probes were made from sorted chromosomes and all 18 peaks in the flow karyotype were identified by same-species FISH. Two peaks each correspond to chromosome pairs, PRO 3, PRO 4, and two peaks to the chromosome pair PRO 9, while the other 14 peaks each correspond to a single pair (PRO 1, 2, 4–8, 10–14, X and Y) (Fig. 2a). The occurrence of the same chromosome in more than one peak usually arises from variations in heterochromatin between homologues.

*Proechimys goeldii* (PGO) has a 2n = 24♀25♂/NFa = 42 karyotype, with the autosomes comprising 10 meta/submetacentric pairs (1–10) and one acrocentric pair (11). The X chromosome is a medium-sized submetacentric, Y_1_ chromosome is a small submetacentric and Y_2_ is a small acrocentric. Same-species FISH identified 11 peaks in the flow karyotype: five correspond to a single chromosome pair (PGO 2, 6, 7, 8 and 9); one corresponds to only a portion of a chromosome (PGO 4q); and five correspond to two chromosomes (PGO [1, 2], [3, X], [4, 5], [10, Y_1_] and [11, Y_2_]) (Fig. 2b).

### Cross-species FISH experiments

We used the PRO probes to establish homologous regions between karyotypes. Multidirectional FISH on *Proechimys* gr. *goeldii* confirmed the exact correspondence of the two probe sets (see Supplementary Figs 1–5). The centromeric regions do not show hybridization signals due to pre-annealing of repeated sequences.

The peaks PGO [3, X], [4, 5], 8, 9 and [11, Y_2_] carry repetitive sequences similar to those found on PGO Y_1_, and thus exhibit signals on the Y and the X (pseudoautosomal region) chromosomes in other karyotypes, even though they do not contain Y chromosome sequences. In the X-autosome translocation of *Proechimys goeldii*, the Y_2_ is homologous to Xp; thus, peak PGO [3, X], which contains the X, also hybridizes to Y_2_ even though it does not contain the Y_2_ chromosome (see Supplementary Figs 1, 3 and 4).

#### PGO probes on PRO metaphases (2n = 30)

**Figure 3:**
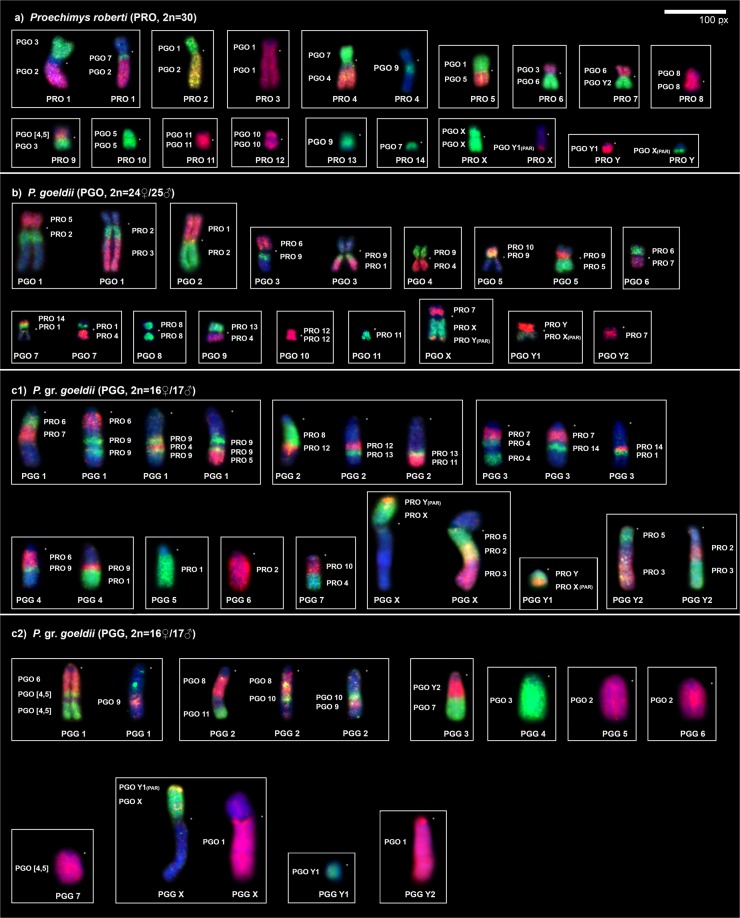
FISH results obtained from (**a**) *Proechimys roberti* (PRO♂), (**b**) *P. goeldii* (PGO♂) and (**c**) *P*. gr. *goeldii* (PGG♂) using the PGO (left) and PRO (right) probes. Each chromosome pair is shown in a box. For some pairs, multiple photos showing different probes are presented to exhibit that the chromosomes were completely covered by the whole-chromosome probes. An asterisk indicates a centromere. Whole-chromosome probes are shown in green (FITC), red (CY3) and yellow (FITC + CY3). Counterstaining is shown in blue (DAPI). Scale bar: 100 pixels.

**Figure 4:**
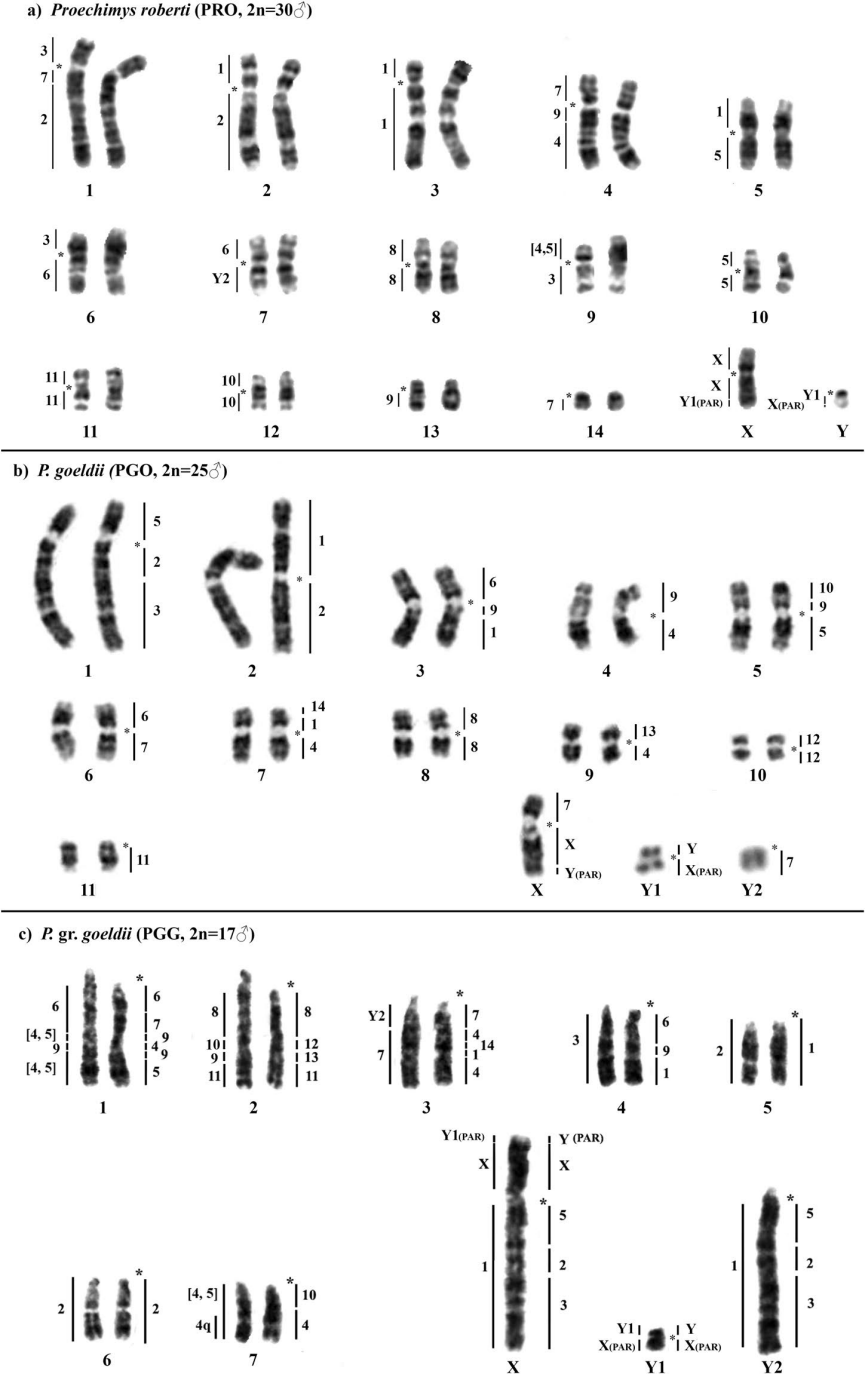
G-banded karyotypes of (**a**) *Proechimys roberti* (PRO♂), (**b**) *P. goeldii* (PGO♂), and (**c**) *P*. gr. *goeldii* (PGG♂). The left and right panels show the PGO and PRO whole-chromosome probes, respectively. An asterisk indicates a centromere.

Cross-species FISH with PGO probes yielded 27 signals on the PRO chromosomes (Table 1, Fig. 3a). Three autosomes are conserved (PGO 8, 10, 11) and hybridize to whole chromosomes of PRO (8, 12 and 11, respectively). The other six show multiple signals on the PRO chromosomes: PGO 2, 6 and 9 hybridize to two chromosomes; PGO 1 and 7 hybridize to three chromosomes each; and PGO [4, 5] hybridize to four chromosomes. Regarding the sex-chromosome probes, PGO X hybridize to PRO X, PRO 7q and Yq distal (pseudoautosomal region - PAR); PGO Y_2_ hybridize to PRO 7q; and PGO Y_1_ hybridize to PRO Yq and Xq distal (PAR). Nine PRO pairs show associations between their syntenic blocks and multiple PGO probes (Fig. 4a). The female karyotype is shown in Supplementary Fig. 6a.

**Table 1:** Chromosomal homology among *Proechimys roberti* (PRO, 2n = 30), *P. goeldii* (PGO, 2n = 24♀25♂) and *P*. gr. *goeldii*^1^ (PGG, 2n = 16♀17♂).

PGO	PRO	PGG	PRO	PGO	PGG
**1**	2p, 3, 5p	Xq, Y_2_	**1**	2p, 3q dist., 7p prox.	3q int., 4q dist., 5
**2**	1q dist., 2q	5, 6	**2**	1q prox., 2q	6, Xq int., Y_2_q int.
**3**	1p, 6p, 9q	4	**3**	1q dist.	Xq dist., Y_2_q dist.
**4q**	—	7q dist.	**4**	4q, 7q, 9q	1q int., 3q int., 3q dist., 7q dist.
**[4, 5]**	4q dist., 5q, 9p, 10	1q int., 1q dist., 7	**5**	1p, 5q	1q dist., Xq prox., Y_2_q prox.
**6**	6q, 7p	1q prox.	**6**	3p, 6p	1q prox., 4q prox.
**7**	1q int., 4p, 14	3q dist.	**7**	6q, Xp, Y_2_	1q int., 3q prox.
**8**	8	2q prox.	**8**	8	2q prox.
**9**	4q prox., 13	1q int., 2q int.	**9**	3q prox., 4p, 5p prox.	1q int. (ts), 4q int.
**10**	12	2q int.	**10**	5p dist.	7q prox.
**11**	11	2q dist.	**11**	11	2q dist.
**X**	X, 7q, Yq dist. (PAR)	Xp, 3q prox., Y_1_q (PAR)	**12**	10	2q int.
**Y**_**1**_	Yq, Xq dist. (PAR)	Y_1_, Xp dist. (PAR)	**13**	9p	2q int.
**Y**_**2**_	7q	3q prox.	**14**	7p dist.	3q int.
			**X**	Xq, Y_1_q (PAR)	Xp, Y_1_q (PAR)
			**Y**	Y_1_, Xq (PAR)	Y_1_, Xp dist. (PAR)

The bold numbers in columns 1 and 4 (from left to right) indicate the PGO and PRO probes, respectively.

PAR (Pseudoautosomal region). Short arm (p). Long arm (q). Proximal (prox.). Interstitial (int.). Distal (dist.). Two segments (ts). ^1^Referred as *Proechimys longicaudatus* by Amaral *et al*.^20^.

#### PRO probes on PGO metaphases (2n = 24♀25♂)

Cross-species FISH with PRO probes yielded 29 signals on the PGO chromosomes (Table 1, Fig. 3b). Seven autosomal probes are conserved; of them, three (PRO 8, 11 and 12) hybridize to whole chromosomes of PGO (8, 11 and 10, respectively) and four (PRO 3, 10, 13 and 14) are associated with portions of other chromosomes (PGO 1q distal, 5p distal, 9p and 7p distal, respectively). Seven autosomal probes show multiple signals in PGO: PRO 2, 5 and 6 hybridize to two chromosomes each, while PRO 1, 4, 7 and 9 hybridize to three chromosomes each. Regarding the sex-chromosome probes, PRO X hybridizes to PGO Xq and Y_1_q (PAR), and PRO Y hybridizes to PGO Y_1_ and Xq distal (PAR). Ten PGO pairs show *Proechimys* associations between their syntenic blocks and various PRO probes (Fig. 4b). The female karyotype is shown in Supplementary Fig. 6b.

#### PRO probes on PGG metaphases (2n = 16♀17♂)

Cross-species FISH with PRO probes yielded 32 signals on the PGG chromosomes (Table 1, Fig. 3c1). Six autosomal probes show whole-chromosome signals with PRO 8, 10, 11, 12, 13 and 14, and also signals on other chromosomes (PGG 2q proximal, 7q proximal, 2q distal, 2q interstitial, 2q interstitial and 3q interstitial, respectively). The other eight autosomal probes show multiple signals in PGG: PRO 3, 6 and 7 hybridize to two chromosomes each, while PRO 1, 2, 4, 5 and 9 hybridize to three chromosomes each. Regarding the sex-chromosome probes, PRO X hybridizes to PGG Xp and PGG Y_1_q (PAR), while PRO Y hybridizes to PGG Y_1_ and PGG Xp distal (PAR). Eight PGG pairs have syntenic blocks that hybridize with multiple PRO probes (Fig. 4c). The female karyotype is shown in Supplementary Fig. 6c.

#### PGO probes on PGG metaphases (2n = 16♀17♂)

Cross-species FISH with PGO probes yielded 20 signals on the PGG chromosomes (Table 1, Fig. 3c2). Six autosomes show conservation: probe PGO 3 hybridizes to the entirety of PGG 4, while probes PGO 6, 7, 8, 10 and 11 are associated with PGG 1q proximal, 3q distal, 2q proximal, 2q interstitial and 2q distal, respectively. Four autosomal probes (PGO 1, 2, [4, 5] and 9) hybridize to two PGG chromosomes each. Probe PGO 4q hybridizes to PGG 7q distal. Regarding the sex-chromosome probes, the PGO X probe hybridizes to PGG Xp, Y_1_q (PAR) and PGG 3q proximal; the PGO Y_1_ probe hybridizes to PGG Y_1_ and Xp distal (PAR); and the PGO Y_2_ probe hybridizes to PGG 3q proximal. Five PGG pairs show associations between their syntenic blocks and multiple PGO probes (Fig. 4c). The female karyotype is shown in Supplementary Fig. 6c.

## Discussion

### Chromosomal rearrangements in *Proechimys* species with the lowest 2n, and signatures for the goeldii group

Although *Proechimys* shows extensive karyotype diversity, with 2n values ranging from 14 to 62, the karyotypes are composed mostly of bi-armed chromosomes^20–22,28^. The sole known exception is an Eastern Amazon *Proechimys* population (PGG), which has an entirely one-armed karyotype (2n = 16♀/17♂)^20^. Here, we have focused our comparative analysis on representatives with low diploid numbers and multiple sex chromosome systems (Fig. 3), namely PRO (2n = 30), PGO (2n = 24♀/25♂) and PGG (2n = 16♀/17♂).

**Figure 5:**
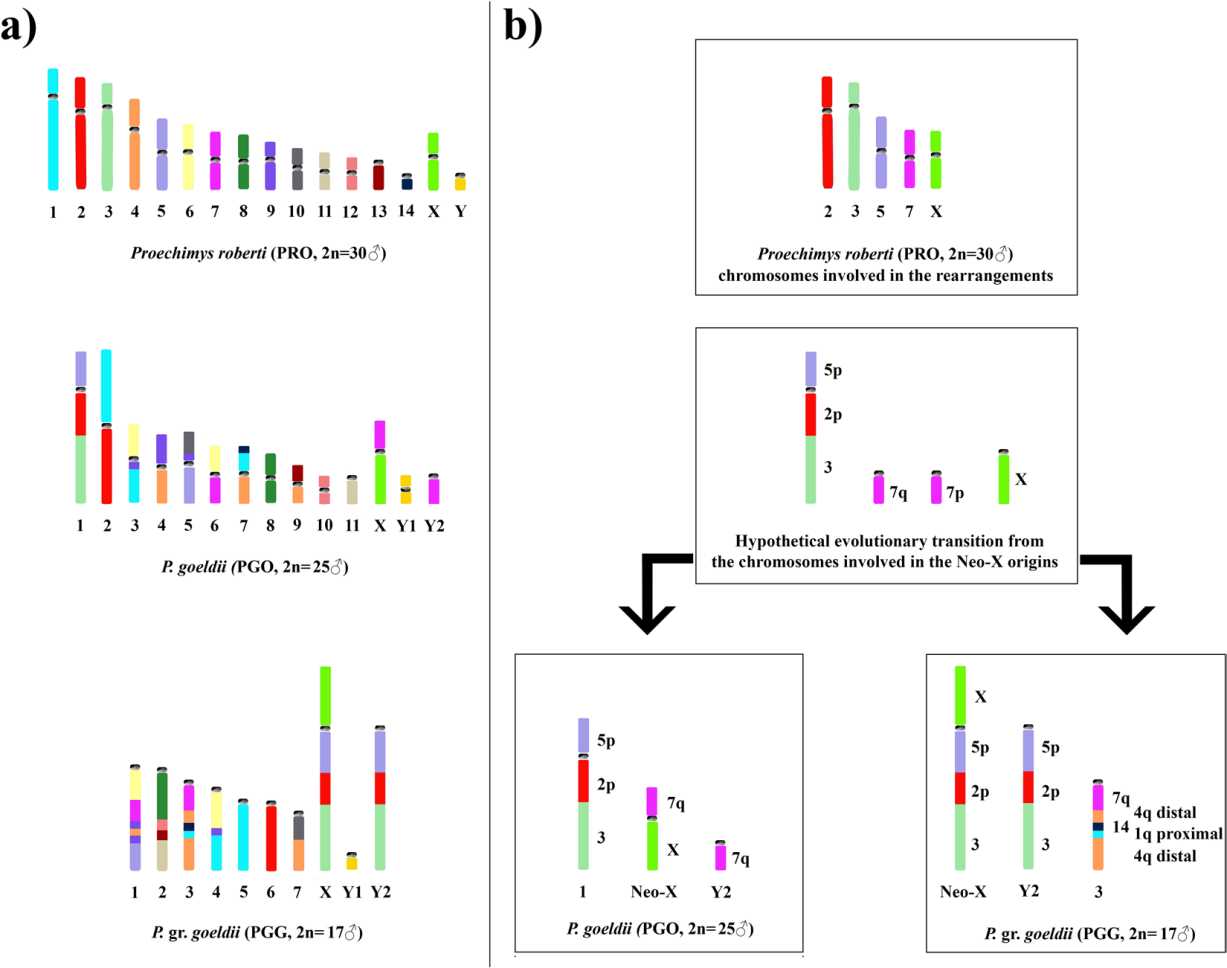
(**a**) Ideograms of the karyotypes of *Proechimys roberti* (PRO♂), *Proechimys goeldii* (PGO♂) and *Proechimys* gr. *goeldii* (PGG♂), as assessed based on the PRO probes. (**b**) Hypothetical evolutionary transition from the chromosomes involved in the X-autosome translocation of PGO and PGG, based on the results obtained using the PRO probes.

Few previous studies have used chromosome banding in *Proechimys* species^29^, and the analyses in the literature have been limited largely to 2n and NFa comparisons. Here, we present the first comparative chromosome painting study for PRO, PGO and PGG. We reveal that these taxa exhibit a high degree of chromosomal variation. We identified two particularly notable patterns. First, between the PRO and PGO karyotypes (2n = 30 and 24♀/25♂, respectively), we detected multiple translocations that largely account for the difference in 2n, and we observed whole-chromosome preservation of only three chromosomes (PRO 8/PGO 8; PRO 12/PGO 10; PRO 11/PGO 11) (Figs 4 and 5a). Second, between PGG and PGO (2n = 24♀/25♂ and 16♀/17♂, respectively), we detected 10 fusion/fission events and two inversions that account for the difference in 2n, and we observed whole-chromosome conservation of eight chromosomes (PRO 5/2/3, 6/9/1, 9/5, 6/7, 14/1/4, 8, 11, and 12) (Figs 4 and 5a).

We propose that these eight chromosomal signatures could be considered as taxonomic/phylogenetic markers for the *goeldii* group. This should assist in their taxonomic identification, since this group has cryptic and/or sympatric species^22,30–32^.

### Causes and implications of the neo-X in Proechimys

The high prevalence of bi-armed chromosomes among the distinct *Proechimys* karyotypes (2n = 14 to 62)^20,21,28^ is shared with other taxa from family Echimyidae, which have diploid numbers ranging from 22 to 118 and a simple sex determining system (XX/XY)^20,21,32–37^. Thus, we propose that the karyotype in PGG with low 2n and all one-armed autosomes is a derived karyotype of the *goeldii* group.

Because the syntenic association “PRO 5/2/3” is shared between PGO 1 (PRO 5/*2/3) and PGG X (PRO X/*/5/2/3), and is distinct from that detected in PGO X (PRO 7/*/X), we propose the following: (1) the syntenic association “PRO 5/2/3” was present in the ancestral karyotype of the *goeldii* group before the diversification events that generated the neo-X in PGG; (2) the bi-armed chromosomal form of PGO 1 indicates that the PGG neo-X chromosome originated from a tandem fusion between the submetacentric autosome and the ancestral acrocentric X, with centromeric inactivation in the translocated autosome; (3) the autosomal segment translocated in PGO X, which is homologous to PGO Y_2_ (PRO */7), is associated with distinct segments of PRO 7 (PGO 6/*/Y_2_) and PGG 3 (PGO */Y_2_/7), indicating that the ancestral form of the translocated segment was an independent chromosome; (4) the neo-X in PGO originated from a Robertsonian translocation between the ancestral acrocentric X and the acrocentric autosome (PRO 7q) (Fig. 5b).

The fixation of an X-autosomal translocation is uncommon in mammals, having been reported in only a few lineages^11,17,38^. This condition can have deleterious effects, as genes on the autosomal portion can be inactivated during the dosage compensation process that occurs in one of the X chromosomes of females; this generally renders the carrier infertile^7^. During male meiosis, the non-homologous XY body undergoes inactivation/condensation^39^, which may spread to the translocated autosomal segment. However, various authors have suggested that this inhibition may be blocked by regions of repetitive DNA, such as heterochromatin^13,27,40,41^ and/or ribosomal DNA sequences^26,42^. In the PGO and PGG species, a significant amount of constitutive heterochromatin separates the autosomal segment from the ancestral X^20,22^, potentially blocking the spread of inactivation from the X segment to the fused autosomal segment.

Our data support the suggestion that the multiple sex chromosome systems (XY_1_Y_2_) observed in the PGO and PGG karyotypes originated independently from each other^22^. This is rare in mammals, in which most X-autosomal translocations are shared among representatives of a lineage^11,14–18,38^. However, such an event was previously reported in rodents of genus *Mus*, subgenus *Nannomys*, in which X-autosome translocations appeared independently^12,18^.

Our unusual observation raises the question as to why multiple systems originated not once but twice in the studied lineage. Farré^43^ proposed that the evolutionary breakpoints are not distributed homogeneously, but instead are concentrated in certain regions of the genome (chromosomal hot spots) that usually have repetitive sequences in their heterochromatin^44^ and are rich in tandem repeats^45^ and/or transposable elements^46^. These features are observed in the PGO and PGG karyotypes, in which the autosome-sex chromosome fusion regions are rich in constitutive heterochromatin and (particularly in PGO) have interstitial telomeric sequences (ITS)^20,22^. Thus, we hypothesize that the ancestral acrocentric X also had numerous repetitive sequences in its pericentromeric/centromeric region (Fig. 5b).

In some mammalian species, certain breakpoints regions have been used multiple times during the evolutionary process^47–49^, without a common ancestry^49^. Therefore, the re-use of a breakpoint region could explain the independent emergence of two neo-X chromosomes in closely related species of genus *Proechimys*.

### Chromosomal differentiation and speciation hypothesis in Proechimys

We herein show that the karyotypes of two closely related species of *Proechimys* (PGO and PGG) are differentiated by multiple chromosomal rearrangements. This karyotypic differentiation can be explained by some biological features of rodents, including: (1) an elevated reproductive rate^50^; (2) a short pregnancy^51^; (3) the birth of a large number of individuals per gestation^34^; and (4) a low vagility^52^. The first three features accelerate the evolutionary process by allowing many generations to be produced in a short period of time. The fourth feature favors endogamy^53^, which increases the likelihood that individuals heterozygous for the rearrangement will interbreed and, within a few generations, form a subpopulation of individuals that are homozygous for the rearranged chromosomal form^7,54–59^.

Chromosomal speciation in sympatry is seen less frequently in animals^6^ than in plants^5^, and we speculate that the emergence of multiple sex chromosomes in a given species could immediately decrease interbreeding between the ancestral (XX/XY) and derived (XY_1_Y_2_) forms, due to the severe problems that would occur during hybrid meiosis. This would agree with the hypothesis that rearrangements could trigger instantaneous speciation through the postzygotic isolation of the ancestral population^60^, without the need for a transitional form^61^. We suggest that this event occurred twice from a common ancestor (XX/XY) to generate the multiple sex chromosome systems in *Proechimys* (Fig. 5b). Alternatively the translocation could cause some morphological or behavioral change leading to a prezygotic isolation mechanism, as seen in the fish, *Gasterosteus aculeatus*^8^.

The sister taxa, PGG and PGO, are sympatric species in the endemic areas of Tapajos/Xingu (Fig. 1) and allopatric species in the endemic areas of Belém (PGO) and Rondonia (PGG)^19,20,22,62,63^. There is no divergence-time data for species of *Proechimys* in the literature; however, the speciation events of genus *Psophia* informed the proposal that the drainage system of the Tapajós River developed approximately 1.3–0.8 Ma^64^. Assuming that an ancestral population for PGG and PGO was distributed in the current endemic areas of Rondonia and Tapajós, the development of the Tapajós River would have acted as a geographic barrier, creating two allopatric subpopulations.

Alternatively, chromosomal rearrangements occurring within subpopulations established in allopatry could play an important role in mediating secondary contact during the geographic expansion of new karyotypic forms. Only strongly isolated neospecies are likely to survive the challenge of sympatry^6^. If weakly isolated, these neospecies may merge through hybridization with their parental population, which would (in general) be more numerous and widely distributed^6^. In this way, chromosomal rearrangements could mediate a rapid speciation process through a post-zygotic blockade of gene flow; the subsequent consolidation of new species, could explain the occurrence of these species in sympatry.

More detailed studies of the center of origin of these two lineages could help elucidate whether this pattern is a typical case of secondary contact between two lineages established in allopatry, or an impressive case of sympatric speciation mediated by chromosomal rearrangements.

In conclusion, our results support the hypothesis that some biological features of Rodentia could explain the fixation of rearrangements in the highly variable karyotypes of *Proechimys* species, and suggest the independent origin of two neo-X chromosomes in *Proechimys* species of group *goeldii*.

## Methods

### Ethics

The specimens were captured using Tomahawk live-traps^65^ and kept stress-free with full access to food and water until their necessary euthanasia was performed in accordance with animal welfare guidelines established by Brazilian resolution CFMV n.1000/2012. The necessary euthanasia occurred in accordance with animal welfare guidelines established by the Animal Ethics Committee (Comitê de Ética Animal) from Universidade Federal do Pará (Permit 68-2015). JCP has a permanent field permit, number 13248 from “Instituto Chico Mendes de Conservação da Biodiversidade”. The Cytogenetics Laboratory from UFPa has a special permit number 19/2003 from the Ministry of Environment for samples transport and 52/2003 for using the samples for research.

### Samples

We studied the karyotypes of *Proechimys roberti* (PRO) and *P. goeldii* (PGO). We analyzed one male and one female from each species, which were acquired from Abaetetuba, Pará state, Brazil (01°39′30″S 48°57′50.02″W). We also examined one male and one female of *Proechimys* gr. *goeldii* (PGG) from Parintins, Amazonas state, Brazil (02°34′45.7″S 56°28′14.4″W) (Fig. 1). Samples were deposited at the zoological collection of Museu de Zoologia da Universidade Federal do Pará (UFPA), Belém, Pará, Brazil.

### Cell culture

Tissue samples obtained from *Proechimys roberti* (2n = 30/NFa = 54), *Proechimys goeldii* (2n = 24♀25♂/NFa = 42) and *Proechimys* gr. *goeldii* (2n = 16♀17♂/NFa = 14) were used to generate cell cultures, as previously described by Morielle-Versute^66^ with adaptations. The genomes of the cultured cells were checked regularly through karyotyping in order to insure that the cell line was stable. Cells were cultured in DMEM supplemented with 15% fetal bovine serum (GIBCO), 2% penicillin (10,000 units/ml) - streptomycin (10,000 μg/ml) (GIBCO) and 2% L-glutamine (200 mM) (GIBCO), and incubated in a CO_2_ incubator at 37 °C. All cell cultures were tested and found to be free of mycoplasma contamination.

### Flow sorting and generation of chromosome-specific probes

Chromosome suspensions were sorted using an adaptation of a previously reported protocol^60^ and a dual-laser cell sorter (MoFlo, Beckman Coulter), as performed at the Cambridge Resource Centre for Comparative Genomics (Cambridge, UK). Chromosome-specific painting probes were made by degenerate oligonucleotide primer PCR (DOP-PCR) amplification of flow-sorted chromosomes^67,68^. DOP-PCR amplified chromosome-specific DNAs were labeled during the secondary PCR by incorporating biotin-16-dUTP (Jena Bioscience) or digoxigenin-11-dUTP (Jena Bioscience). The PRO and PGO painting probes were generated as previously described^69^.

### Cytogenetics

Chromosomal preparations were obtained by fibroblast cell culture of skin biopsies (see above), which was performed at the Centro de Estudos Avançados da Biodiversidade, Instituto de Ciências Biológicas, Universidade Federal do Pará, Brazil and the Resource Centre for Comparative Genomics. The chromosomal preparations were G-banded^70^. Whole-chromosome probes of PRO and PGO were used for FISH experiments, following a procedure adapted from Yang^69^ We omitted the use of DNA salmon sperm and mouse Cot-1 DNA, and instead performed pre-annealing of repetitive sequences^71^.

### Image capture and processing

Digital images were captured using the Zeiss AXIOPLAN 2 microscope with Metasystems ISIS version 5.4, or Nikon H550S microscopy with Nis-Elements software.

### Map

The map was made using QUANTUM-GIS (QGIS) program version 2.10.1. Database was obtained from DIVA, IBGE and REDLIST (Fig. 1).

## Supplementary information

Table 1
