# Supplementary material for: Identification of two independent X-autosome translocations in closely related mammalian (Proechimys) species
Source: Sci Rep. 2019 Mar 11;9:4047. doi: 10.1038/s41598-019-40593-8 (PMC6411977; doi:10.1038/s41598-019-40593-8)
Supplement: Supplementary file 1 — Table 1 [file 41598_2019_40593_MOESM1_ESM.pdf]

Identification of two independent X-autosome translocations in closely related mammalian (*Proechimys*) species

Willam Oliveira da Silva, Marlyson Jeremias Rodrigues da Costa, Julio Cesar Pieczarka, Jorge Rissino, Jorge C. Pereira, Malcolm Andrew Ferguson-Smith, Cleusa Yoshiko Nagamachi

Supplementary Table 1. List of the *Proechimys roberti* (PRO), *P. goeldii* (PGO) and *P.* gr. *goeldii* (PGG) samples karyotyped in the present study and from the literature, with sample collection points and coordinates indicated. The numbers refer to the localities mentioned in Figure 1. The noted Brazilian (BR) states are Amazonas (AM), Pará (PA) and Mato Grosso (MT).

Species	Locality	Reference
PRO, PGO	(1) BR, PA: Abaetetuba (01°39’30”S 48°57’50.02”W)	Present study
PGO	(2) BR, PA: Tailândia (02º32’58”S 48º43’55”W)	Rodrigues da Costa et al.[15]
PGO	(3) BR, PA: Mocajuba (02º37’20”S 49º30’31”W)	Rodrigues da Costa et al.[15]
PGO	(4) BR, PA: Marabá (05°48’5”S 50°30’54”W)	Rodrigues da Costa et al.[15]
PGO	(5) BR, PA: Altamira (03°26’57”S 52°17’31”W)	Patton et al.[25]
PGO	(6) BR, PA: Belterra (03º02’43”S 54°55’59”W)	Rodrigues da Costa et al.[15]
PGG	(7) BR, AM: Parintins (02°34’45”S 56°28’14”W)	Present study
PGO, PGG	(8) BR, PA: Itaituba (05°14’7.78”S 56°55’52”W)	Rodrigues da Costa et al.[15]
PGO, PGG	(9) BR, PA: Jacareacanga (06°7’16”S 57°34’49”W)	Rodrigues da Costa et al.[15]
PGG	(10) BR, MT: Cotriguaçu (09°51’17”S 58°14’53”W)	Amaral et al.[13]
PGG	(11) BR, MT: Juruena (10°27’8.74”S 58°39’46”W)	Machado et al.[12]
PGO	(12) BR, MT: Alta Floresta (09°37’13”S 55°54’49”W)	Patton et al.[25]
PGG	(13) BR, MT: Querência (12°00’54”S 52°00’22”W)	Amaral et al.[13]

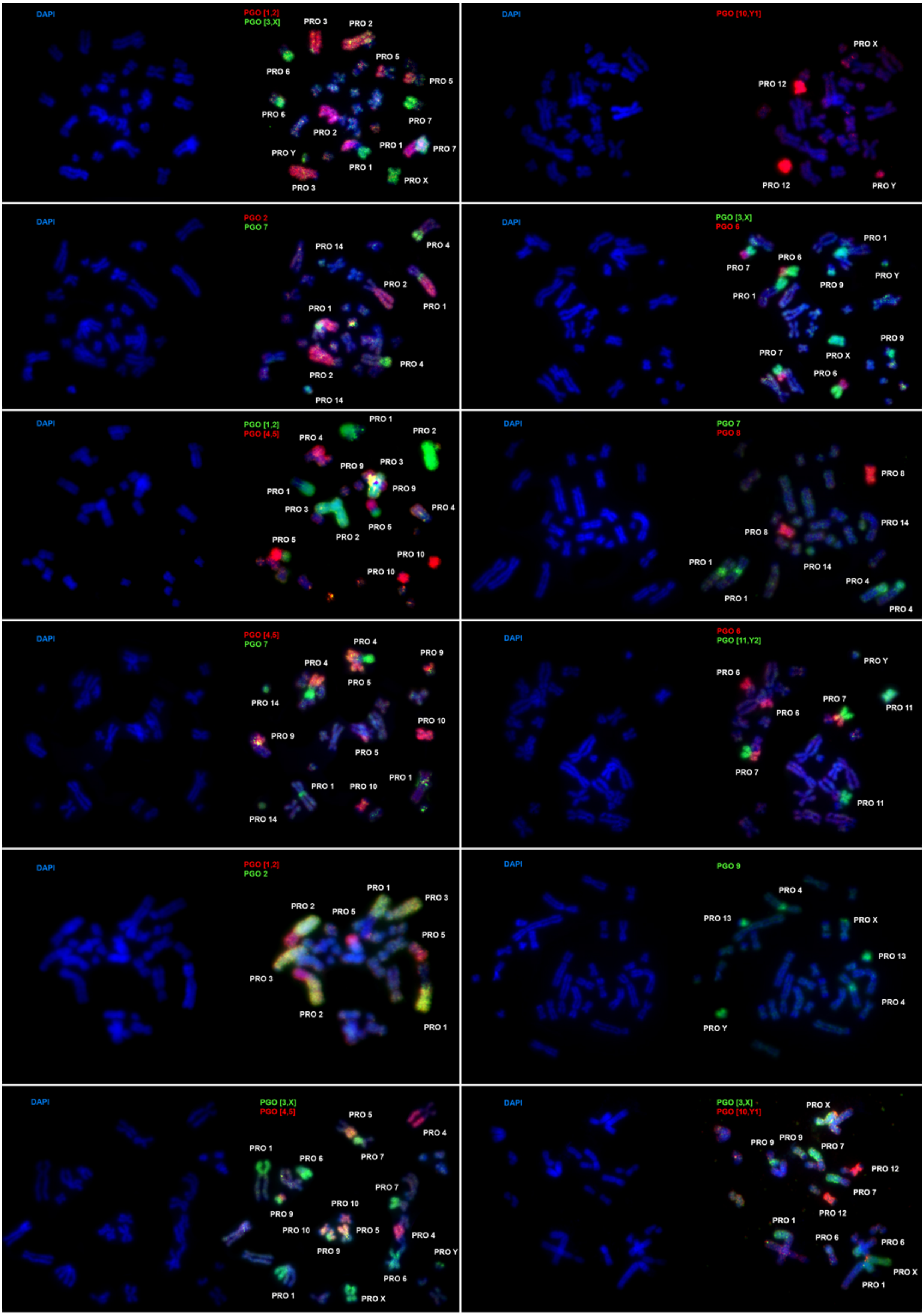

Supplementary Figure 1. FISH of *Proechimys roberti* (PRO) metaphases with *P. goeldii* (PGO) probes. For each metaphase, the probe composition and color is noted (top right), while the same metaphase is shown with DAPI staining on the left.

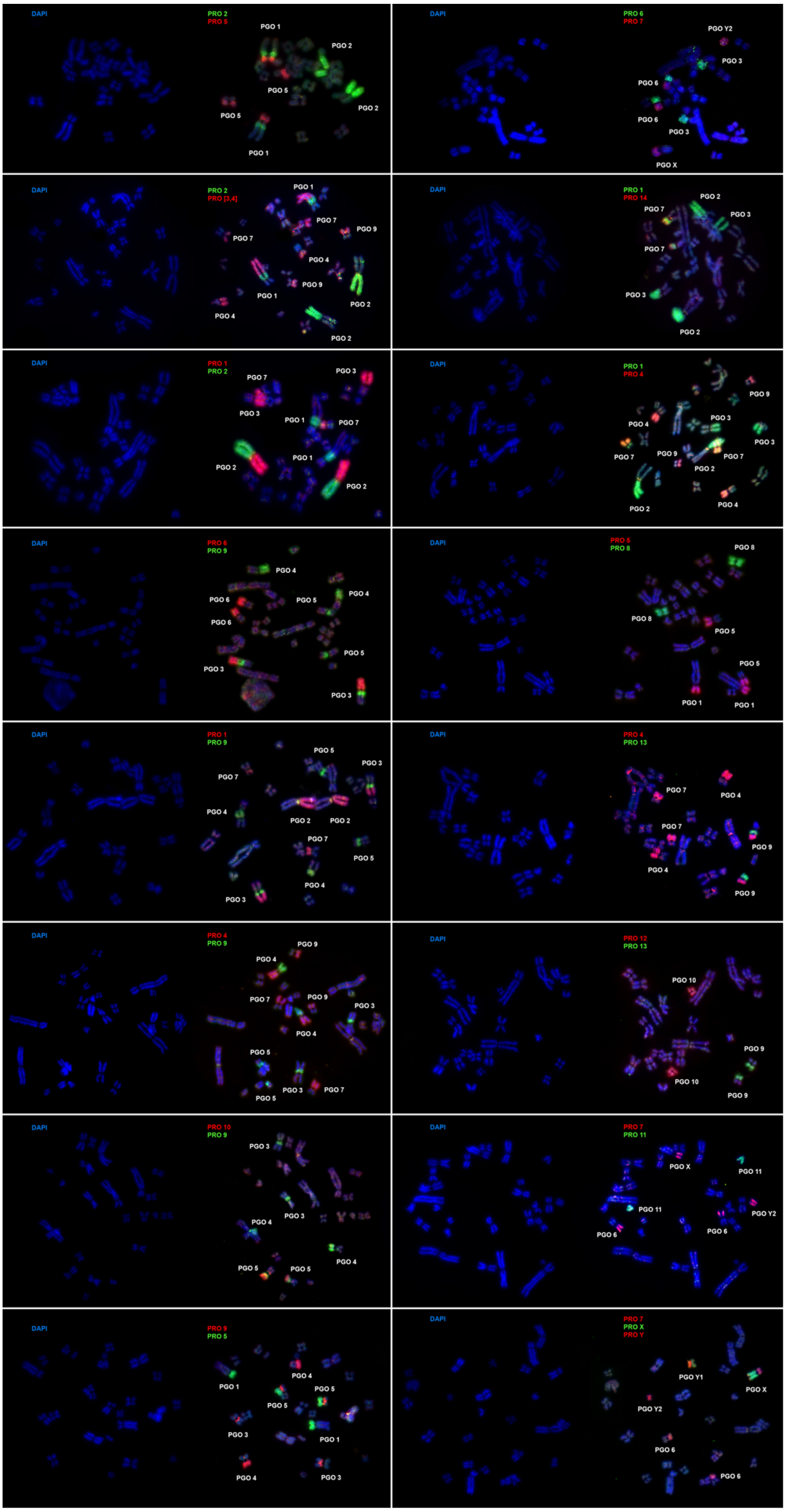

Supplementary Figure 2. FISH of *Proechimys goeldii* (PGO) metaphases with *P. roberti* (PRO) probes. For each metaphase, the probe composition and color is noted (top right), while the same metaphase is shown with DAPI staining on the left.

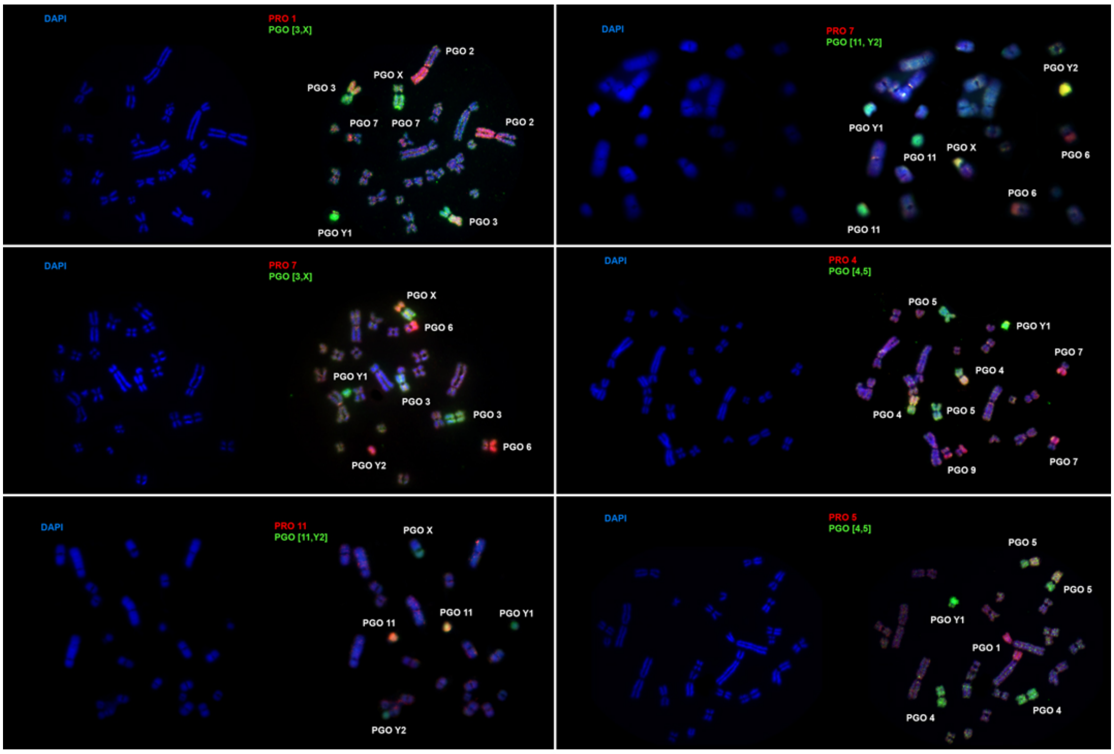

Supplementary Figure 3. FISH of *Proechimys goeldii* metaphases with *Proechimys roberti* (PRO) and *Proechimys goeldii* (PGO). For each metaphase, the probe composition and color is noted (top right), while the same metaphase is shown with DAPI staining on the left.

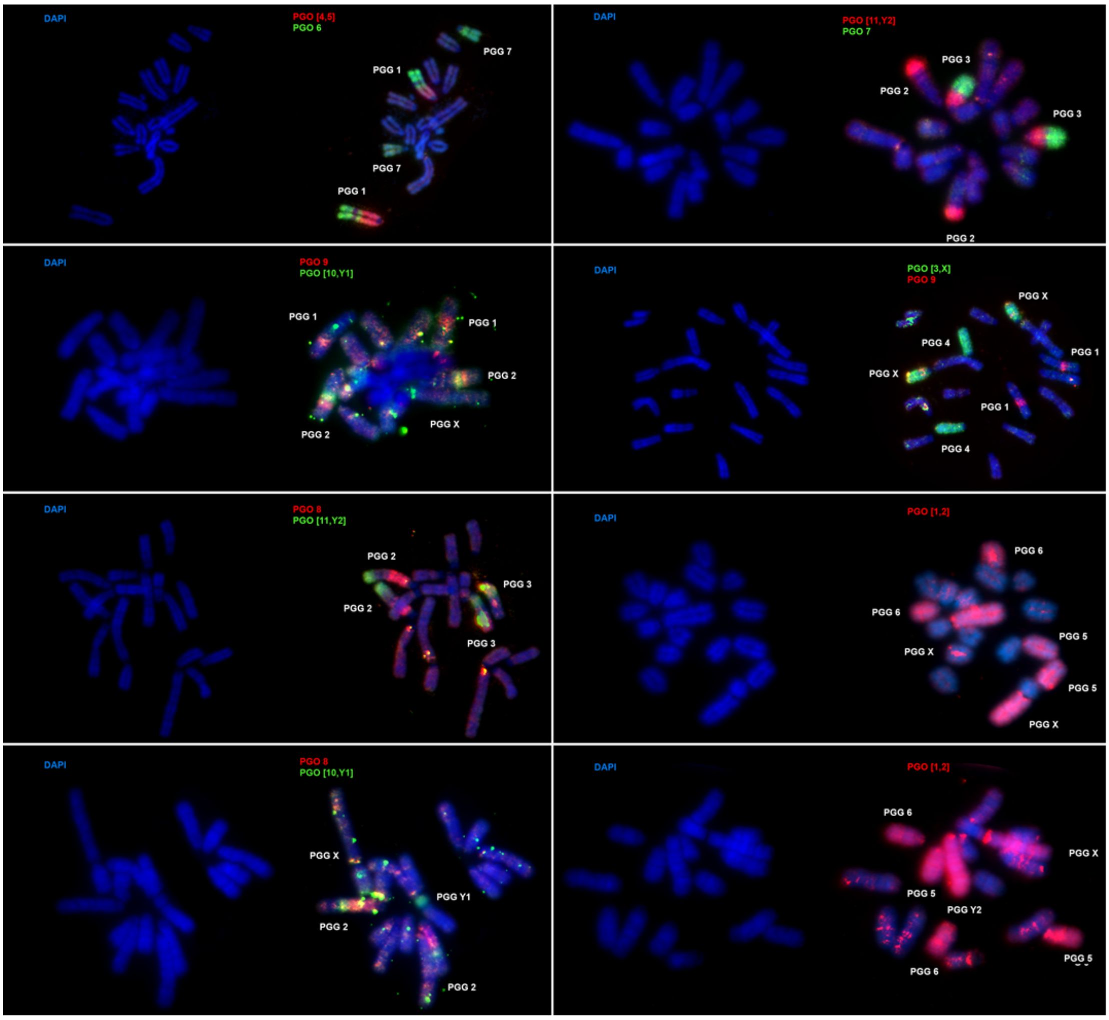

Supplementary Figure 4. FISH of *Proechimys* gr. *goeldii* (PGG) metaphases with *P. goeldii* (PGO) probes. For each metaphase, the probe composition and color is noted (top, right), while the same metaphase is shown with DAPI staining on the left.

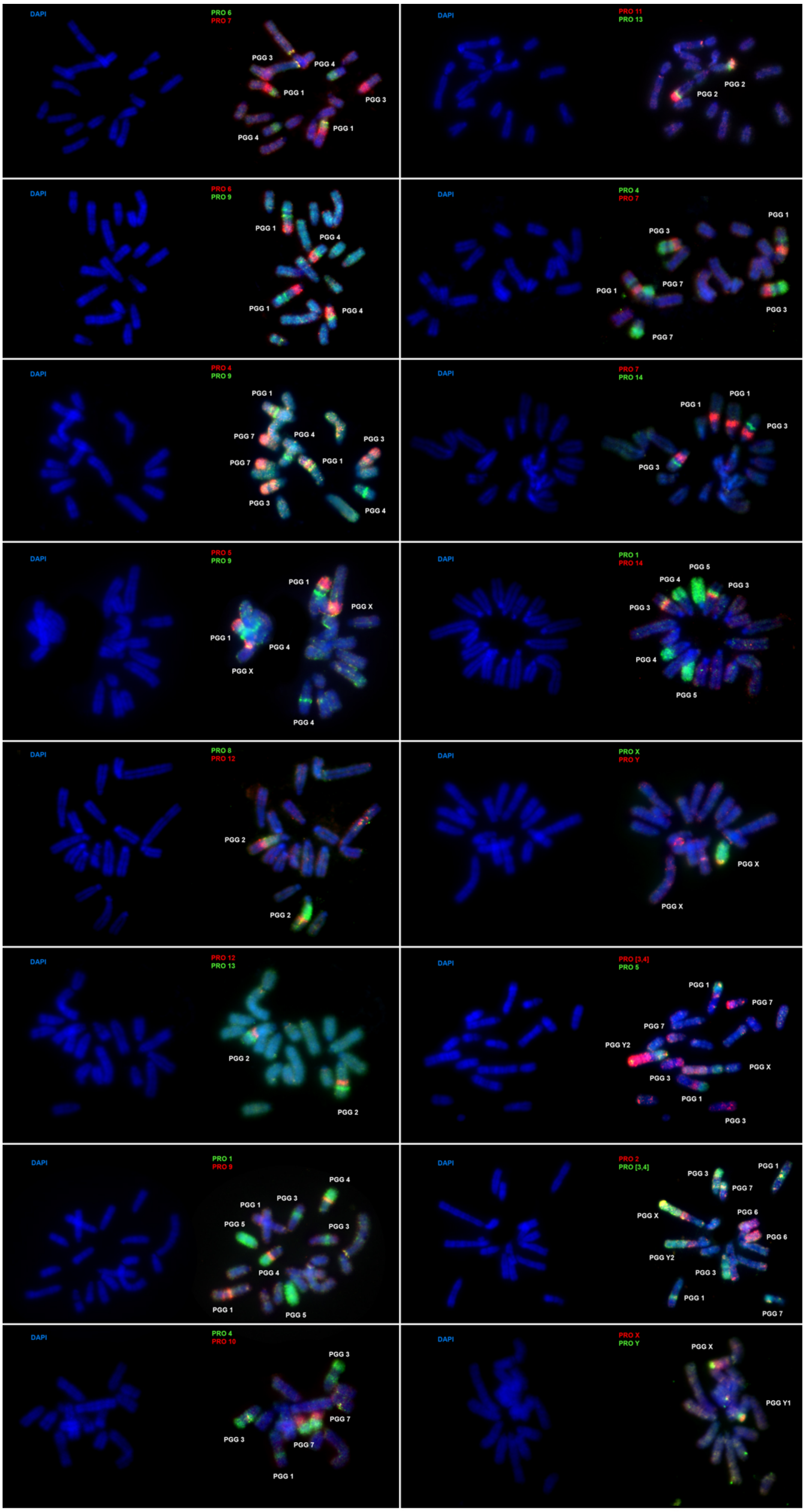

Supplementary Figure 5. FISH of *Proechimys* gr. *goeldii* (PGG) metaphases with *P. roberti* (PRO) probes. For each metaphase, the probe composition and color is noted (top, right), while the same metaphase is shown with DAPI staining on the left.

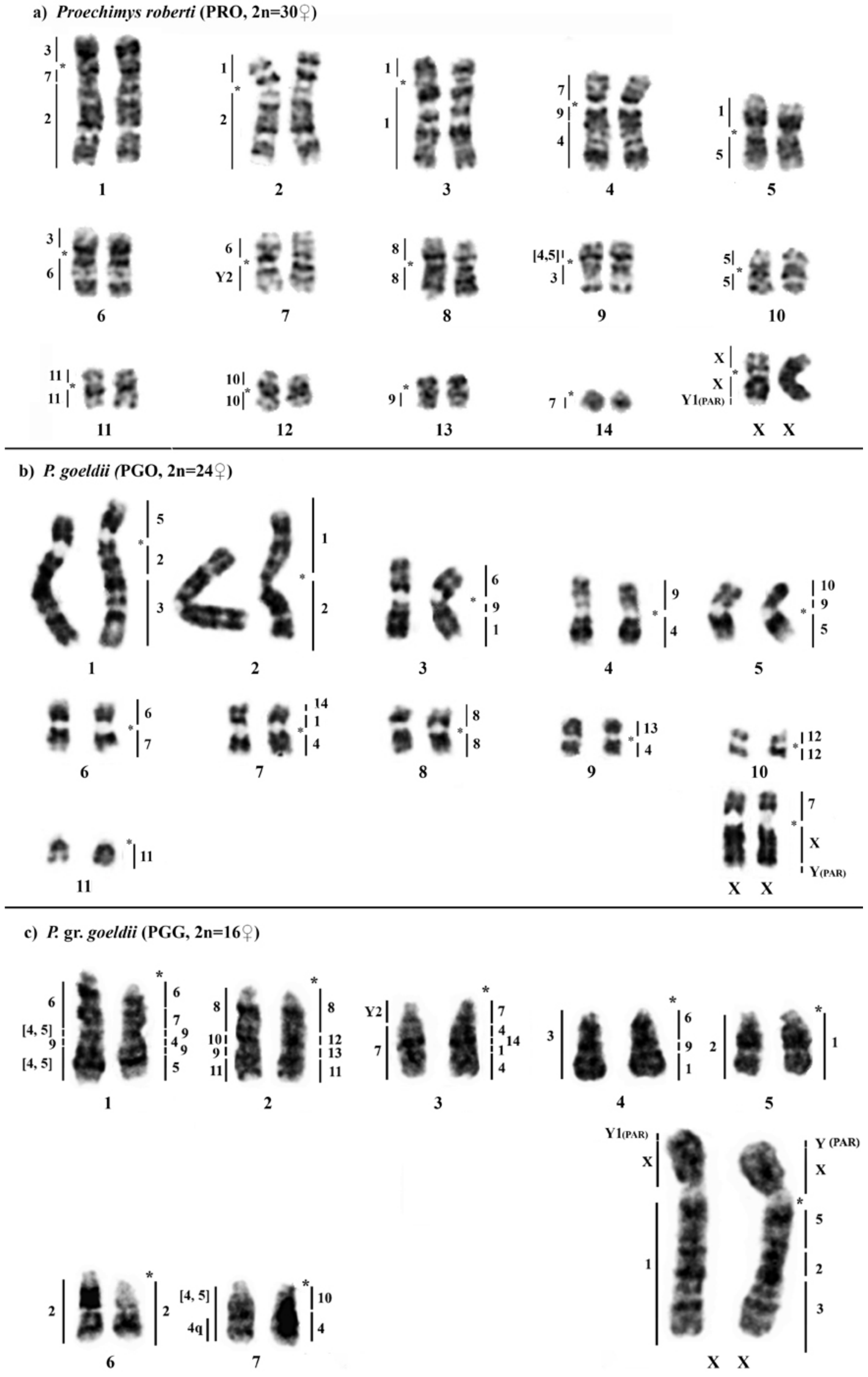

Supplementary Figure 6. G-banded karyotypes of a) *Proechimys roberti* (PRO♀), b) *P. goeldii* (PGO♀) and c) *P.* gr. *goeldii* (PGG♀) with chromosome painting performed using the PGO (left) and PRO (right) probes. An asterisk indicates a centromere.
